# Explainable deep-learning models to predict diaphragmatic dysfunction and cognitive stress in ICU patients under mechanical ventilation

**DOI:** 10.3389/fphys.2026.1765898

**Published:** 2026-04-02

**Authors:** Yonghua Wang, Yuling Bai, Ge Jin

**Affiliations:** 1 Respiratory Intensive Care Unit, The First Affiliated Hospital of Zhengzhou University, Zhengzhou, China; 2 The First Affiliated Hospital of Zhengzhou University, Zhengzhou, China

**Keywords:** cognitive stress/delirium, deep learning, diaphragmatic dysfunction, lung–brain axis, mechanical ventilation, multimodal prediction, ultrasound

## Abstract

**Background:**

Diaphragmatic dysfunction and acute cognitive stress/delirium are serious complications of mechanical ventilation that prolong intensive care unit (ICU) stay and are associated with increased mortality. Although accumulating evidence suggests a potential lung–brain axis linking impaired respiratory muscle function to adverse neurocognitive trajectories, early risk stratification remains challenging because clinically relevant signals are multimodal, temporally dynamic, and often partially observed. We aimed to develop an interpretable multimodal deep learning model to predict diaphragmatic dysfunction and high cognitive stress/delirium in mechanically ventilated patients and to identify shared predictors that may reflect lung–brain crosstalk.

**Methods:**

We conducted a multicenter retrospective study including 25,751 mechanically ventilated ICU patients. A multimodal long short-term memory (LSTM) network was trained using continuous clinical time-series variables (vital signs, ventilator parameters, and medication dosing trajectories) and diaphragm ultrasound videos from a sub-cohort (n = 4,783). Discrimination was evaluated using the area under the receiver operating characteristic curve (AUC) and the area under the precision–recall curve (AUPRC). Post-hoc SHapley Additive exPlanations (SHAP) were applied to quantify feature contributions and explore cross-modal interactions.

**Results:**

In the independent test set, the multimodal model outperformed both clinical-only (AUC = 0.811) and video-only (AUC = 0.749) baselines for predicting diaphragmatic dysfunction, achieving an AUC of 0.902 (95% CI, 0.88–0.92) and an AUPRC of 0.594 (both P < 0.001 vs. baselines). For high cognitive stress/delirium prediction, the model achieved an AUC of 0.792. Calibration was acceptable, with close agreement between predicted and observed risks (Brier score = 0.12). SHAP analyses indicated that ultrasound-derived diaphragm thickening fraction (DTF) and cumulative neuromuscular blockade exposure were among the strongest predictors of diaphragmatic dysfunction. Notably, diaphragm excursion and heart rate variability emerged as shared influential predictors for cognitive stress/delirium, consistent with the lung–brain crosstalk hypothesis in mechanically ventilated patients.

**Conclusion:**

This study presents a multimodal deep learning framework for early identification of mechanically ventilated patients at elevated risk of diaphragmatic dysfunction and high cognitive stress/delirium. Integrating diaphragm ultrasound with longitudinal bedside physiologic and treatment data improves prediction beyond single-modality models, while post hoc explainability highlights candidate shared predictors relevant to a lung–brain axis. These findings suggest that integrated monitoring of respiratory mechanics and sedation-related physiology may support more proactive ventilator management and neuroprotective ICU care.

## 1 Introduction

Mechanical ventilation is a cornerstone life-sustaining intervention in intensive care units (ICUs). However, its downstream complications—particularly diaphragmatic dysfunction and neurocognitive sequelae—are increasingly recognized as substantial “hidden costs” that prolong ICU stay and worsen outcomes. Diaphragmatic dysfunction has been reported in 60%–80% of critically ill patients receiving mechanical ventilation, with major contributors including disuse atrophy due to diaphragm unloading, systemic inflammation, and medication-related neuromuscular junction injury. These impairments are consistently associated with weaning failure, prolonged ventilation, and higher ICU mortality (Bose et al., 2022; De Trizio et al., 2025). Clinically, approximately one in five ventilated patients experience difficulty in liberation, and the weaning phase itself may account for nearly 40% of total ventilation time, creating a sustained burden on ICU resources and patient recovery (De Trizio et al., 2025).

In parallel, delirium and acute cognitive stress are frequent in the ICU and can carry long-lasting consequences. Evidence from systematic reviews and large cohort studies indicates that ICU delirium is associated with prolonged mechanical ventilation, extended ICU and hospital stays, increased healthcare costs, higher in-hospital and post-discharge mortality, and long-term cognitive decline (Wang et al., 2025). Notably, a substantial proportion of patients who experience ICU delirium report persistent memory problems after discharge (Wang et al., 2025). Current preventive and management strategies typically combine conventional weaning evaluation, bedside diaphragm ultrasound assessment, and delirium screening programs such as CAM-ICU and RASS, complemented by non-pharmacological measures including early mobilization, sleep optimization, and environmental interventions (Bandyopadhyay et al., 2023).

Despite their widespread use, existing tools remain constrained in complex ICU settings. Diaphragm ultrasound measurements—particularly diaphragm thickening fraction (DTF) and indices of diaphragmatic motion—have been explored to estimate readiness for liberation from mechanical ventilation (Sali et al., 2025; Fonseca et al., 2024). However, systematic reviews and cohort studies have reported substantial heterogeneity in threshold selection, measurement timing, and the operational definition of weaning failure, resulting in variable sensitivity and specificity and limited generalizability across settings (Zhou Q. et al., 2025; Sepúlveda et al., 2025). Ultrasound assessment is also operator-dependent and sensitive to image quality, which complicates standardization and real-time quantification, especially in older patients and those with multi-organ dysfunction or complex ventilator modes (Pan et al., 2024). More fundamentally, diaphragmatic function cannot be fully understood in isolation: successful spontaneous breathing reflects a coupled system involving respiratory mechanics, cardiovascular interactions, sedation and analgesia exposure, and systemic physiological stress. Meanwhile, prediction models for ICU delirium or cognitive impairment have been proposed, but most rely on traditional statistical approaches or a small set of static variables, and therefore struggle to capture time-varying drivers such as cumulative sedative exposure, evolving ventilator settings, and dynamic inflammatory burdens (Marques et al., 2024; Contreras et al., 2025; Yang et al., 2025). In routine practice, diaphragmatic dysfunction risk and cognitive stress/delirium are often evaluated separately, leaving a gap in integrated assessment of how these processes may co-evolve within the same patient over time.

The increasing availability of electronic health records and high-frequency bedside monitoring has accelerated the use of machine learning and deep learning for ICU outcome prediction. Prior work has shown that recurrent architectures (RNN, GRU, and LSTM) can effectively model irregular, multivariate ICU time-series and learn temporal dependencies that are difficult to represent with static features, enabling prediction of outcomes such as in-hospital mortality, prolonged length of stay, and readmission risk (Sadaf et al., 2023). These sequence models are particularly well-suited to ICU monitoring because key exposures and physiological responses accumulate over time and their clinical meaning often depends on trajectory rather than single measurements. Interpretable machine learning approaches have also been applied to ICU outcomes using methods such as gradient-boosted trees and graph learning, highlighting the importance of temporal resolution and patient-specific feature attribution. Building on these developments, multimodal learning has begun to enter respiratory support decision-making: multimodal frameworks that combine diaphragm ultrasound with clinical indicators have outperformed single ultrasound parameters in predicting mechanical ventilation disconnection failure, and models integrating ultrasound from multiple organs further underscore the value of multimodal information in complex physiological prediction tasks (Asmare et al., 2025).

Nevertheless, important limitations remain. Most existing studies focus on a single outcome and do not jointly model functional complications that frequently co-occur, such as diaphragmatic dysfunction and cognitive stress/delirium. In terms of data modalities, many approaches are restricted to structured clinical variables and summary ultrasound parameters, with limited use of the spatiotemporal patterns embedded in diaphragm ultrasound videos. Regarding interpretability, feature-importance analyses are sometimes reported, but few studies systematically examine which diaphragmatic, ventilatory, and sedation-related variables jointly drive respiratory deterioration and cognitive stress across different time windows in the same patient. This limitation is particularly relevant for evaluating the proposed “lung–brain axis” in mechanically ventilated populations, where shared predictors and time-dependent interactions are central.

To address these clinical and methodological gaps, we developed a multimodal deep learning framework to predict diaphragmatic dysfunction and high cognitive stress/delirium in mechanically ventilated ICU patients. The framework integrates heterogeneous sources including continuous vital signs, ventilator parameters, medication dosing trajectories, relevant clinical scores and assessments, and diaphragm ultrasound videos. Sequential modeling is performed using recurrent time-series networks, while a dedicated ultrasound video encoder captures spatiotemporal features beyond handcrafted measurements; these representations are fused to enable joint prediction within a unified learning framework. Model interpretability is provided via post-hoc explainability using SHapley Additive exPlanations (SHAP), which quantifies feature contributions and highlights candidate shared predictors across outcomes, with the goal of supporting earlier risk stratification and more proactive optimization of ventilator management, sedation/analgesia strategies, and neuroprotective ICU care.

The remainder of this paper is organized as follows: the Methods section describes the cohort, data modalities, model architecture, training procedure, and evaluation strategy; the Results section reports predictive performance, calibration, and explainability findings; the Discussion section interprets the clinical implications and limitations; and the Conclusion summarizes key contributions and future directions.

## 2 Materials and methods

### 2.1 Study design and population

**FIGURE 1:**
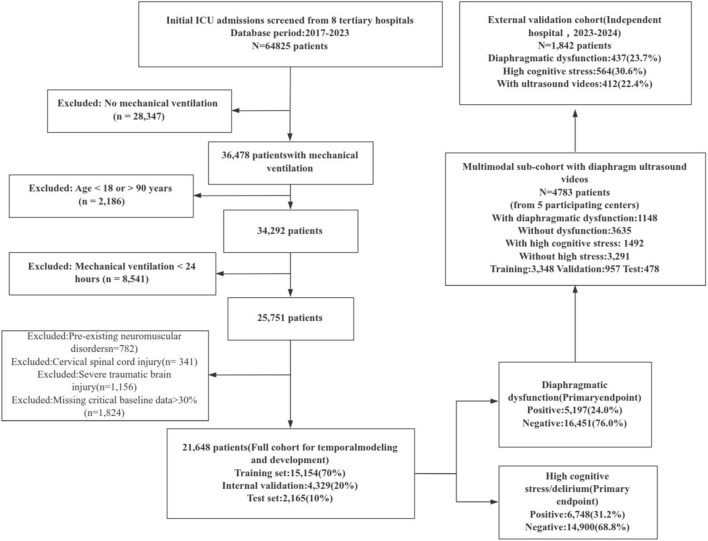
Flow diagram of cohort construction, data partitioning, and outcome-group allocation for mechanically ventilated ICU patients.

This multicenter retrospective cohort study was conducted using electronic health record (EHR) data from ICUs at eight tertiary hospitals to develop the prediction models, with external validation performed in an independent tertiary hospital (Figure 1). The development cohort covered admissions from 2017 to 2023. Adult ICU admissions were screened from the EHR systems (n = 64,825) and were progressively filtered using predefined criteria: (i) exclusion of patients without mechanical ventilation (n = 28,347), leaving 36,478 ventilated patients; (ii) exclusion of patients aged <18 years or >90 years (n = 2,186), leaving 34,292; (iii) exclusion of short-term ventilation (<24 h) (n = 8,541), leaving 25,751; and (iv) exclusion of patients with prior neuromuscular disorders (n = 782), cervical spinal cord injuries (n = 341), severe traumatic brain injuries (n = 1,156), or >30% missing critical baseline data (n = 1,824). The final analytic “full cohort” included 21,648 patients for time-series modeling and model development.

For model development, the full cohort was randomly split into training (n = 15,154; 70%), internal validation (n = 4,329; 20%), and test (n = 2,165; 10%) sets (Figure 1). To build and evaluate the multimodal model, five of the eight hospitals with long-term standardized diaphragm ultrasound monitoring protocols contributed a multimodal sub-cohort with diaphragm ultrasound videos (n = 4,783). In this sub-cohort, 1,148 patients had diaphragmatic dysfunction and 3,635 did not; 1,492 patients had high cognitive stress/delirium and 3,291 did not. The multimodal sub-cohort was randomly split into training (n = 3,348; 70%), validation (n = 957; 20%), and test (n = 478; 10%) sets.

External validation was conducted using an independent cohort enrolled from 2023 to 2024 (n = 1,842 mechanically ventilated ICU patients). In this cohort, 437 patients (23.7%) had diaphragmatic dysfunction, 564 (30.6%) had high cognitive stress/delirium, and 412 (22.4%) had available diaphragm ultrasound videos. The two shared primary endpoints were (i) diaphragmatic dysfunction and (ii) high cognitive stress/delirium. In the full cohort, 5,197 patients (24.0%) were positive for diaphragmatic dysfunction and 16,451 (76.0%) were negative; 6,748 (31.2%) were positive for high cognitive stress and 14,900 (68.8%) were negative.

### 2.2 Outcomes and definitions

Two primary outcomes were defined: diaphragmatic dysfunction and high cognitive stress/delirium. A composite adverse outcome was defined as the secondary endpoint.

1. Diaphragmatic dysfunction. Standardized diaphragm ultrasound examinations were performed under stable ventilator conditions by trained bedside ultrasound physicians or certified critical care physicians. Bilateral diaphragm thickness and motion were assessed during tidal breathing and deep inspiration. Diaphragm thickening fraction (DTF) and diaphragm excursion (range of motion) were calculated. Consistent with prior literature and clinical practice, diaphragmatic dysfunction was defined as DTF <20% on either side or diaphragm excursion <10 mm on either side; no dysfunction was assigned if both sides met thresholds. For patients with repeated measurements, the ultrasound results within the 24 h prior to the clinical decision to discontinue mechanical ventilation were used for modeling.
2. High cognitive stress/delirium. Consciousness and delirium were assessed at least once daily during ICU stay using the Richmond Agitation–Sedation Scale (RASS) and Confusion Assessment Method for the ICU (CAM-ICU). High cognitive stress/delirium was defined as persistent or recurrent CAM-ICU positivity (≥2 positive assessments) at any time during mechanical ventilation, or CAM-ICU positivity accompanied by |RASS| ≥2 requiring additional pharmacological intervention. All remaining cases were classified as the non-high cognitive stress group. To minimize interference from residual drug effects, time windows under deep sedation (RASS −4 to −5) during which meaningful cognitive assessment was not feasible were excluded from outcome evaluation.

Composite adverse outcome (secondary endpoint). To relate functional outcomes to “hard outcomes,” the composite adverse outcome was defined as any of the following: failure of liberation from mechanical ventilation, ICU death, or ICU length of stay exceeding the upper quartile threshold of the cohort. Any occurrence of these conditions was counted as a composite adverse outcome.

### 2.3 Multimodal data sources and preprocessing

Four categories of data were constructed.

1. Physiologic and ventilator time-series. High-frequency monitoring data were automatically extracted at 1–5 min intervals, including heart rate, invasive/non-invasive blood pressure, respiratory rate, SpO_2_, temperature, and ventilator variables (e.g., tidal volume, PEEP, FiO_2_, ventilator mode, pressure support level).
2. Laboratory results and severity scores. Routine laboratory tests (e.g., blood gas variables, lactate, inflammatory markers, electrolytes, hepatic and renal function indices) and severity scores (e.g., APACHE II, SOFA) were collected. Timestamps were aligned to the monitoring timeline.
3. Cognitive and sedation-related data. RASS, CAM-ICU, sedative/analgesic categories, and dosing trajectories were mapped to a continuous timeline using administration timestamps, enabling modeling of cumulative exposure and dynamic changes.
4. Diaphragm ultrasound videos. Bilateral B-mode and M-mode ultrasound videos were collected from centers with standardized protocols. Videos were converted to a uniform frame rate and spatial resolution, and clips containing complete respiratory cycles were extracted.

Preprocessing. Time-series variables were resampled to a uniform grid and segmented using sliding windows. Missing values were handled using forward filling and linear interpolation when clinically appropriate, with explicit missingness indicators appended to preserve information about unobserved measurements. Extreme outliers were truncated or Winsorized prior to z-score normalization for continuous features. Static demographic variables and baseline comorbidities were incorporated as non-time-varying covariates.

Handling modality availability and ultrasound video heterogeneity. Because diaphragm ultrasound videos were not available for all patients or time points, multimodal model training and evaluation were performed within the predefined ultrasound sub-cohort (n = 4,783) with paired videos and aligned physiologic/EHR time-series. For patients with multiple ultrasound examinations, we selected the video clip acquired closest to (and prior to) the predefined clinical decision window used for modeling (e.g., within 24 h before ventilator discontinuation decisions for diaphragmatic outcomes). To accommodate variable clip duration, videos were converted to a fixed frame rate, and clips were sampled/padded to a fixed length with a corresponding mask to indicate valid frames, ensuring consistent input dimensionality for the video encoder.

### 2.4 Temporal modeling architectures: RNN, GRU, and LSTM

**FIGURE 2:**
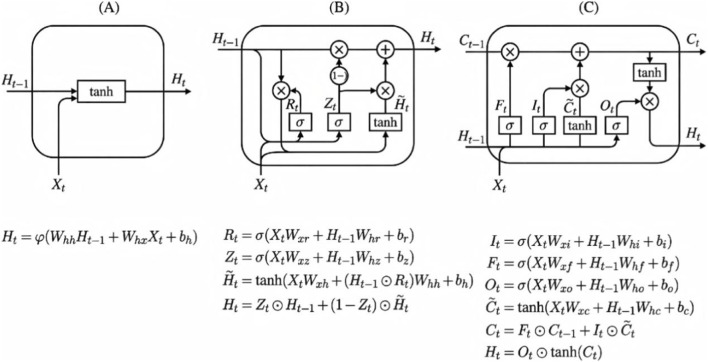
Recurrent units used for temporal feature extraction: **(A)** RNN, **(B)** GRU, **(C)** LSTM.

ICU monitoring data exhibit strong temporal dependence and exposure accumulation effects (e.g., ventilator adjustments, sedative dosing trajectories, and evolving physiologic responses). Therefore, we evaluated three recurrent architectures as temporal feature extractors: RNN, GRU, and LSTM (Figure 2). A basic RNN updates hidden states based on the current input and the previous hidden state and is effective for short-range dependence but can suffer from vanishing/exploding gradients in long sequences. GRU introduces reset and update gates to improve gradient flow with fewer parameters. LSTM maintains an explicit cell state controlled by input/forget/output gates and is widely used to capture longer-range dependencies in complex medical time-series.

For each patient, multivariate time-series data within a predefined observation window were represented as a T × F input matrix, where T is the number of resampled time steps within the observation window and F is the number of features per time step (including physiologic variables, ventilator parameters, and aligned clinical trajectories). The matrix was fed into the respective recurrent unit (RNN/GRU/LSTM) with stacked layers and dropout regularization to obtain a temporal latent representation. This representation was later fused with ultrasound video features and static covariates for downstream prediction. To ensure a fair comparison, models were trained under identical data partitioning and hyperparameter search strategies, and predictive performance for diaphragmatic dysfunction, high cognitive stress/delirium, and composite adverse outcome was evaluated on independent test sets.

### 2.5 Multimodal explainable deep-learning framework

**FIGURE 3:**
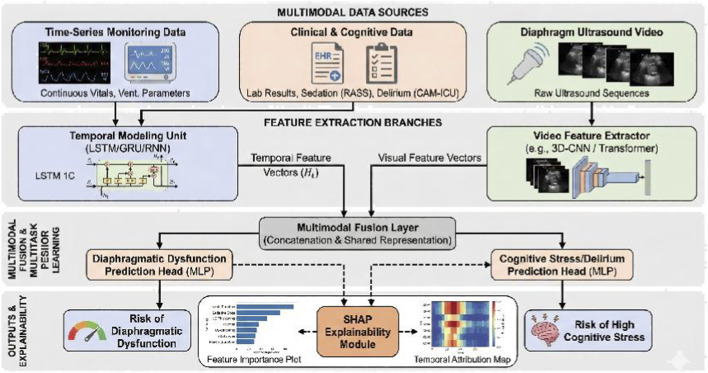
Overview of the multimodal multi-task framework. Inputs include physiologic/ventilator time-series $X^{ts}$ , aligned EHR-derived trajectories $X^{ehr}$ (laboratory values, severity scores, sedative/analgesic dosing trajectories, RASS/CAM-ICU), diaphragm ultrasound videos $X^{vid}$ , and static covariates $X^{stat}$ . Outputs include predicted probabilities of diaphragmatic dysfunction ${\hat{p}}^{dia}$ and high cognitive stress/delirium ${\hat{p}}^{cog}$ . Post-hoc SHAP is applied after model training to quantify feature contributions and visualize temporal attributions across modalities.

An overview of the proposed framework is shown in Figure 3. The model consists of four components: (i) data inputs, (ii) modality-specific feature extraction, (iii) multimodal fusion, and (iv) multi-task prediction with post-hoc explainability.

Inputs. The framework consumes three aligned inputs: (1) physiologic and ventilator time-series from monitoring systems; (2) EHR-derived clinical variables including laboratory values, organ function scores, sedative/analgesic dosing trajectories, and cognitive/delirium assessments (RASS, CAM-ICU); and (3) diaphragm ultrasound video sequences. Static covariates (demographics/comorbidities) were included as non-time-varying inputs.

Feature extraction. The time-series branch uses a recurrent network (RNN/GRU/LSTM) to generate a temporal feature vector. Ultrasound videos are encoded using a dedicated video encoder (e.g., 3D-CNN or video Transformer) to capture spatiotemporal visual patterns beyond summary measurements. Static clinical and laboratory covariates are embedded using a multi-layer perceptron (MLP).

Fusion. Modality-specific vectors are integrated into a shared representation via concatenation or attention-based weighting to capture cross-modal interactions between diaphragmatic function, systemic physiology, ventilatory mechanics, and cognitive load.

Multi-task outputs. Two task-specific heads are attached to the shared representation: a diaphragmatic dysfunction prediction head and a high cognitive stress/delirium prediction head. Each head outputs a risk probability, enabling joint learning of related functional outcomes within a unified model.

Explainability (post-hoc). Model interpretability is provided via post-hoc explainability using SHapley Additive exPlanations (SHAP) to quantify feature contributions for each prediction, rather than an inherently interpretable model form. SHAP analyses were conducted at both global and patient-specific levels, and time-window aggregation was applied to derive temporal attribution profiles for dynamic variables.

### 2.6 Model training, internal validation, and evaluation metrics

All models were trained using the development cohort training set, with early stopping based on internal validation performance, and final evaluation performed on the held-out test set and the external validation cohort. Binary cross-entropy loss was used for both tasks. Optimization was performed using Adam with an initial learning rate of 1 × 10^−3^, with learning rate adjustment driven by validation loss. Mini-batch gradient descent was used with a batch size of 32–64. Early stopping was triggered when validation AUC did not improve over consecutive epochs to mitigate overfitting. To address class imbalance, class-weighted loss was applied, and focal loss was additionally evaluated in sensitivity analyses. Model stability was assessed using 5-fold cross-validation within the development cohort.

Performance metrics. Discrimination was assessed using AUC and AUPRC. Additional metrics included accuracy, sensitivity, specificity, F1 score, and Brier score. Receiver operating characteristic curves, precision–recall curves, and calibration curves were generated to evaluate discrimination and calibration.

### 2.7 Explainability analysis using SHAP

To improve clinical interpretability of the final selected multimodal model, SHAP was used to decompose each predicted probability into additive contributions from input features. SHAP values were computed for all patients in the internal test set and the external validation cohort to obtain (i) global feature importance rankings and effect directions for both outcomes, and (ii) patient-level explanations for individual risk predictions.

For time-series variables, SHAP values were aggregated within sliding windows to generate temporal attribution heatmaps, highlighting how evolving vital signs, ventilator parameters, and sedation depth contributed to risk over time. For ultrasound-derived and static clinical features, summary plots (e.g., swarm and bar plots) were used to visualize average contributions and identify potentially modifiable predictors. Representative cases were selected to illustrate local explanations at clinically relevant time points, supporting clinician-facing interpretation of high-risk alerts.

### 2.8 Pseudocode of the multimodal training and inference pipeline (code not open-sourced)

To enhance reproducibility without releasing source code, we summarize the computational workflow and formalize the model inputs, fusion representation, and multi-task objective. For each patient, structured bedside monitoring and ventilator trajectories were resampled onto a uniform grid to form a multivariate sequence $X^{ts}\in R^{T\times F_{ts}}$ . Time-stamped EHR-derived trajectories—including laboratory values, severity scores, medication administrations (both contemporaneous dosing and cumulative exposure features), and cognition-related assessments—were aligned to the same grid, yielding $X^{ehr}\in R^{T\times F_{ehr}}$ . Static covariates such as demographics and baseline comorbidities were represented as $X^{stat}\in R^{F_{stat}}$ . Missingness in structured variables was addressed using clinically appropriate forward filling and interpolation, while explicit missing indicators were retained as additional features to preserve information about partially observed measurements; continuous variables were normalized after outlier handling.

For the imaging modality, diaphragm ultrasound clips were standardized to a fixed frame rate and spatial resolution. Because clip duration varies across examinations, each clip was sampled/padded to a fixed length L, producing $X^{vid}\in R^{L\times H\times W\times C}$ , together with a validity mask $M^{vid}$ to ensure padded frames do not contribute to representation learning. When multiple ultrasound examinations were available for a patient, the clip closest to (and prior to) the predefined clinical decision window used for modeling was selected to maximize temporal relevance to the outcome definition.

Feature extraction was performed in a modality-specific manner. The structured temporal streams were encoded by a recurrent temporal encoder (LSTM as the primary model, with RNN/GRU evaluated under identical protocols for comparison) to produce a temporal representation $h^{ts}=f_{ts}\left(\left[X^{ts};X^{ehr}\right],X^{stat}\right)$ . Ultrasound videos were encoded using a dedicated spatiotemporal video encoder to capture motion-related patterns beyond handcrafted summary indices, yielding $h^{vid}=f_{vid}\left(X^{vid},M^{vid}\right)$ . If a separate static encoder was used, we obtained $h^{stat}=f_{stat}\left(X^{stat}\right)$ . These modality-specific representations were integrated into a shared embedding via concatenation or attention-based weighting:

$$\backslash z=\text{Fuse}\left(h^{ts},h^{vid},h^{stat}\right)$$

Downstream prediction was formulated as a multi-task learning problem with two task-specific heads operating on the shared embedding:

$${\hat{p}}^{dia}=\sigma \left(g_{dia}\left(z\right)\right),\;{\hat{p}}^{cog}=\sigma \left(g_{cog}\left(z\right)\right)$$

where ${\hat{p}}^{dia}$ denotes the predicted probability of diaphragmatic dysfunction and ${\hat{p}}^{cog}$ denotes the predicted probability of high cognitive stress/delirium.

Model training optimized a weighted multi-task objective to account for class imbalance and to jointly learn both outcomes:

$$L=\lambda_{dia}\text{ BCE}\left(y^{dia},{\hat{p}}^{dia}\right)+\lambda_{cog}\text{ BCE}\left(y^{cog},{\hat{p}}^{cog}\right)$$

with class-weighted binary cross-entropy as the primary loss and focal loss evaluated in sensitivity analyses. Optimization was performed using Adam with mini-batch training, dropout regularization, and early stopping guided by internal validation AUC to reduce overfitting. Final performance was evaluated on held-out test sets and the external validation cohort using discrimination (AUC/AUPRC) and calibration metrics.

Interpretability was addressed post hoc after model training. SHapley Additive exPlanations (SHAP) were computed on the finalized multimodal model to quantify feature contributions at both global and patient-specific levels. For dynamic variables, SHAP values were aggregated over sliding windows to derive temporal attribution profiles, enabling visualization of how evolving physiology, ventilator settings, and sedation depth contributed to predicted risk trajectories. For ultrasound-derived and static inputs, summary visualizations were used to characterize the magnitude and direction of feature effects, supporting clinician-facing interpretation of high-risk predictions.

## 3 Results

### 3.1 Baseline characteristics of the study cohort

Baseline characteristics of the 25,751 mechanically ventilated ICU patients are summarized in Table 1. Stratified analyses by outcomes showed that patients who developed diaphragmatic dysfunction (Outcome 1) and those who developed high cognitive stress/delirium (Outcome 2) were older than their respective negative groups (mean age >68 years in positive groups vs. <62 years in negative groups; all P < 0.001). Adverse outcome groups also had higher proportions of emergency admissions and inter-hospital transfers (all P < 0.001), suggesting greater illness acuity at presentation.

**TABLE 1:** Baseline characteristics of mechanically ventilated ICU patients stratified by diaphragmatic dysfunction and cognitive stress outcomes.

Characteristic	Total (N = 25,751)	Outcome 1: Diaphragmatic dysfunction			Outcome 2: High cognitive stress			Outcome 3: Composite adverse outcome		
		Positive (n = 6,180)	Negative (n = 19,571)	P	Positive (n = 7,983)	Negative (n = 17,768)	P	Positive (n = 8,240)	Negative (n = 17,511)	P
Age (mean ± SD)	63.6 ± 16.2	68.9 ± 14.8	61.9 ± 16.5	<0.001	70.4 ± 13.9	60.5 ± 16.8	<0.001	71.8 ± 15.2	59.8 ± 15.9	<0.001
Sex, n (%)										
Male	14,960 (58.1%)	3,844 (62.2%)	11,116 (56.8%)	<0.001	4,287 (53.7%)	10,673 (60.1%)	<0.001	5,043 (61.2%)	9,917 (56.6%)	<0.001
Female	10,791 (41.9%)	2,336 (37.8%)	8,455 (43.2%)		3,696 (46.3%)	7,095 (39.9%)		3,197 (38.8%)	7,594 (43.4%)	
Ethnicity, n (%)										
White	16,890 (65.6%)	4,091 (66.2%)	12,799 (65.4%)	0.235	5,205 (65.2%)	11,685 (65.8%)	0.381	5,455 (66.2%)	11,435 (65.3%)	0.142
Black american	3,120 (12.1%)	723 (11.7%)	2,397 (12.2%)		974 (12.2%)	2,146 (12.1%)		972 (11.8%)	2,148 (12.3%)	
Asian	1,460 (5.7%)	358 (5.8%)	1,102 (5.6%)		439 (5.5%)	1,021 (5.7%)		453 (5.5%)	1,007 (5.8%)	
Hispanic	2,450 (9.5%)	606 (9.8%)	1,844 (9.4%)		782 (9.8%)	1,668 (9.4%)		808 (9.8%)	1,642 (9.4%)	
Admission location, n (%)										
Emergency room	16,900 (65.6%)	4,524 (73.2%)	12,376 (63.2%)	<0.001	5,971 (74.8%)	10,929 (61.5%)	<0.001	6,196 (75.2%)	10,704 (61.1%)	<0.001
Physician referral	3,350 (13.0%)	519 (8.4%)	2,831 (14.5%)		599 (7.5%)	2,751 (15.5%)		643 (7.8%)	2,707 (15.5%)	
Transfer from hospital	3,500 (13.6%)	1,082 (17.5%)	2,418 (12.4%)		1,357 (17.0%)	2,143 (12.1%)		1,327 (16.1%)	2,173 (12.4%)	
Others	1,000 (3.9%)	55 (0.9%)	945 (4.8%)		56 (0.7%)	944 (5.3%)		74 (0.9%)	926 (5.3%)	
LOS (days, mean ± SD)	10.3 ± 4.1	17.8 ± 8.4	8.9 ± 3.6	<0.001	15.4 ± 7.2	8.8 ± 3.5	<0.001	20.1 ± 9.8	8.5 ± 3.2	<0.001
Metastatic cancer, n (%)										
Yes	3,500 (13.6%)	1,421 (23.0%)	2,079 (10.6%)	<0.001	1,676 (21.0%)	1,824 (10.3%)	<0.001	2,142 (26.0%)	1,358 (7.8%)	<0.001
No	22,250 (86.4%)	4,759 (77.0%)	17,491 (89.4%)		6,307 (79.0%)	15,944 (89.7%)		6,098 (74.0%)	16,153 (92.2%)	
Hematologic malignancy, n (%)										
Yes	1,100 (4.3%)	445 (7.2%)	655 (3.3%)	<0.001	495 (6.2%)	605 (3.4%)	<0.001	684 (8.3%)	416 (2.4%)	<0.001
No	24,650 (95.7%)	5,735 (92.8%)	18,916 (96.7%)		7,488 (93.8%)	17,163 (96.6%)		7,556 (91.7%)	17,095 (97.6%)	
Aids, n (%)										
Yes	300 (1.2%)	99 (1.6%)	201 (1.0%)	0.002	128 (1.6%)	172 (1.0%)	<0.001	173 (2.1%)	127 (0.7%)	<0.001
No	25,451 (98.8%)	6,081 (98.4%)	19,370 (99.0%)		7,855 (98.4%)	17,596 (99.0%)		8,067 (97.9%)	17,384 (99.3%)	

Comorbidity burden differed substantially across outcome strata. For example, metastatic cancer was more prevalent among patients with composite adverse outcomes (Outcome 3) (26.0% vs. 7.8% in the negative group; P < 0.001). Across all outcomes, adverse groups were associated with increased resource utilization, reflected by significantly prolonged ICU length of stay (LOS), including a mean LOS of 20.1 ± 9.8 days in the composite adverse outcome–positive group (P < 0.001).

### 3.2 Diaphragm ultrasound sub-cohort and multimodal data availability

The multimodal sub-cohort included 4,783 patients with paired diaphragm ultrasound videos and synchronized clinical time-series data, comprising more than 4.2 million standardized ultrasound frames. Imaging metadata and quantitative diaphragm measurements are summarized in Table 2.

**TABLE 2:** Overview of demographics, clinical outcomes, and diaphragm ultrasound video characteristics in the multimodal cohort.

Characteristics	Total cohort (N = 4,783)	Dysfunction group (n = 1,148)	Non-dysfunction group (n = 3,635)	P-value
Demographics and clinical baseline				
Age (years), mean ± SD	64.2 ± 15.8	69.5 ± 14.1	62.5 ± 16.0	<0.001
BMI (kg/m^2^), mean ± SD	24.6 ± 4.5	24.2 ± 4.1	24.8 ± 4.6	0.082
APACHE II score	18.5 ± 6.2	24.1 ± 7.5	16.7 ± 4.8	<0.001
Duration of MV before ultrasound (days)	4.5 ± 2.8	6.8 ± 3.5	3.8 ± 1.9	<0.001
Ultrasound video metadata				
Total video Clips, n	14,349	3,444	10,905	
Total frames analyzed, n (millions)	4.2	1.1	3.1	
Clip duration (seconds), mean ± SD	8.8 ± 2.4	9.2 ± 2.6	8.6 ± 2.3	<0.001
Frame rate (fps), median [IQR]	30 [25–40]	30 [25–40]	30 [25–40]	0.456
Resolution >640 × 480, n (%)	3,826 (80.0%)	918 (80.0%)	2,908 (80.0%)	0.982
Image quality score (1–5 scale)*	4.2 ± 0.8	3.9 ± 0.9	4.3 ± 0.7	<0.001
Quantitative diaphragm metrics				
B-mode features (morphology)				
End-expiratory thickness (tee), mm	1.9 ± 0.6	1.4 ± 0.4	2.1 ± 0.6	<0.001
End-inspiratory thickness (tei), mm	2.5 ± 0.8	1.6 ± 0.5	2.8 ± 0.7	<0.001
Thickening fraction (DTF), %	28.5 ± 12.4	16.2 ± 4.5	32.4 ± 11.2	<0.001
Mean pixel intensity (echogenicity)†	45.2 ± 15.6	68.4 ± 18.2	37.9 ± 10.4	<0.001
M-mode features (mechanics)				
Diaphragm excursion (DE), mm	14.5 ± 5.6	8.2 ± 3.1	16.5 ± 5.2	<0.001
Inspiratory velocity, mm/s	12.8 ± 4.2	8.5 ± 3.6	14.1 ± 3.8	<0.001
Clinical outcomes				
Weaning failure, n (%)	1,052 (22.0%)	689 (60.0%)	363 (10.0%)	<0.001
High cognitive stress/Delirium, n (%)	1,492 (31.2%)	643 (56.0%)	849 (23.4%)	<0.001
ICU mortality, n (%)	861 (18.0%)	402 (35.0%)	459 (12.6%)	<0.001

Overall video quality was suitable for deep-learning–based feature extraction. Although the diaphragmatic dysfunction group had slightly lower image quality scores (3.9 ± 0.9 vs. 4.3 ± 0.7; P < 0.001), 80.0% of videos across both groups met or exceeded VGA resolution (≥640 × 480). Quantitative ultrasound measurements demonstrated expected functional differences between groups, including lower DTF and reduced diaphragm excursion in the dysfunction group (both P < 0.001). The dysfunction group also exhibited higher mean pixel intensity (echogenicity) (68.4 ± 18.2 vs. 37.9 ± 10.4; P < 0.001), indicating that pixel-intensity–based descriptors may carry additional imaging-derived information beyond conventional mechanical indices, in the present work, echogenicity is treated as a predictive feature rather than being used to infer a specific tissue pathology.

### 3.3 Model configuration and experimental environment

To facilitate reproducibility, we report the full computational environment and training configuration used to generate the results. All models were implemented in Python 3.10 with PyTorch 2.1.0 and CUDA 11.8, running on Ubuntu 20.04 LTS. Experiments were executed on a single-GPU workstation equipped with an NVIDIA RTX 3090 (24 GB VRAM), an Intel Xeon Silver 4210 CPU, and 128 GB RAM. Core dependencies included NumPy (1.26), SciPy (1.11), scikit-learn (1.3), and the SHAP library (0.44). To ensure determinism, we fixed random seeds for Python/NumPy/PyTorch (seed = 2023) and enabled deterministic cuDNN settings where supported; all reported metrics were produced from the fixed data splits described in Section 2.

Across experiments, models were trained using the Adam optimizer with an initial learning rate of 1 \times 10^{-3}, and learning-rate reduction was triggered by plateauing validation loss (factor 0.5, patience 3). Mini-batch training used batch size = 64 for time-series models and batch size = 32 for the multimodal model due to video memory requirements. Early stopping was applied based on internal validation AUC with patience = 10 epochs, and training was capped at 100 epochs. Gradient clipping (global norm = 5.0) was used to stabilize optimization for long sequences. Class imbalance was handled via class-weighted binary cross-entropy in the primary analysis, and focal loss (γ = 2) was evaluated as a sensitivity analysis with consistent conclusions. Model stability was further assessed using 5-fold cross-validation within the development cohort under the same hyperparameter search space.

For the time-series branch (RNN/GRU/LSTM), multivariate physiologic and ventilator trajectories were resampled to a uniform grid (5-min intervals) and represented as fixed-length sequences with T time steps and F features as specified in Section 2.4. We used a 2-layer LSTM (hidden size = 128, dropout = 0.3) as the main temporal encoder, with analogous capacity-matched RNN and GRU baselines for fair comparison. Static covariates were embedded using a multilayer perceptron (MLP; 2 hidden layers of 128 and 64 units, ReLU activations, dropout 0.2). For the ultrasound branch, diaphragm videos were standardized to 30 fps and normalized in spatial resolution; frames were center-cropped around the diaphragm region and resized to 224 × 224 for encoding. Each training sample used a fixed-length clip (32 frames) sampled from a complete respiratory cycle. Video features were extracted using a 3D-CNN encoder (3D-ResNet-18 backbone), and fused with time-series and static embeddings via concatenation followed by an MLP fusion block (128 units). Two task-specific prediction heads output probabilities for diaphragmatic dysfunction and high cognitive stress/delirium. Calibration curves and Brier scores were computed from held-out test predictions without recalibration unless otherwise stated.

Post-hoc explainability was performed using SHAP on the final selected multimodal model. For deep components, SHAP values were estimated using a gradient-based SHAP variant (Deep SHAP) with a background set sampled from the training data. For time-series inputs, SHAP attributions were aggregated over predefined sliding windows to produce temporal attribution profiles, while ultrasound-derived features and static covariates were summarized using global swarm and bar plots. All baseline and multimodal models were trained and evaluated under identical data partitions and matching optimization protocols to ensure that performance differences reflected modeling choices rather than hardware or training-procedure discrepancies.

### 3.4 Performance of recurrent models across tasks

**FIGURE 4:**
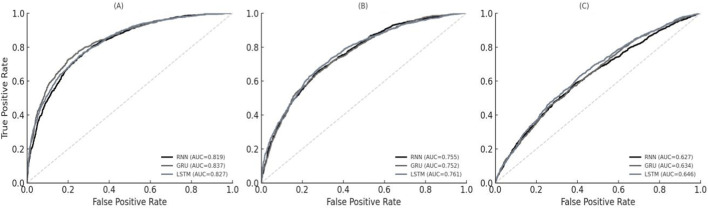
Receiver operating characteristic (ROC) curves comparing RNN, GRU, and LSTM models in the independent test set: **(A)** diaphragmatic dysfunction, **(B)** high cognitive stress/delirium, and **(C)** composite adverse outcome.

In the independent test set (n = 2,165), we evaluated three recurrent architectures (RNN, GRU, and LSTM) using clinical time-series monitoring data alone (Table 3; Figure 4). Overall, LSTM consistently achieved the strongest performance across all three outcomes, with significantly higher AUC and AUPRC than the reference RNN model (all P < 0.001), and generally superior performance to GRU.

**TABLE 3:** Performance of recurrent neural network models (RNN, GRU, LSTM) in predicting diaphragmatic dysfunction, high cognitive stress, and composite adverse outcomes in ICU patients.

Outcome and model	AUC (95% CI)	AUPRC (95% CI)	Sensitivity	Specificity	PPV	NPV	F1-score	P-value (vs. RNN)
Outcome 1: Diaphragmatic dysfunction								
RNN (reference)	0.764 (0.742–0.785)	0.452 (0.410–0.495)	0.685	0.724	0.442	0.881	0.537	
GRU	0.812 (0.792–0.831)	0.528 (0.485–0.570)	0.742	0.765	0.501	0.902	0.598	<0.01
LSTM	0.845 (0.826–0.863)	0.594 (0.551–0.636)	0.789	0.792	0.548	0.921	0.647	<0.001
Outcome 2: High cognitive stress								
RNN (reference)	0.715 (0.692–0.738)	0.385 (0.345–0.425)	0.624	0.698	0.395	0.854	0.484	
GRU	0.758 (0.736–0.779)	0.462 (0.420–0.505)	0.695	0.731	0.448	0.886	0.545	0.035
LSTM	0.792 (0.771–0.812)	0.515 (0.472–0.558)	0.736	0.758	0.492	0.905	0.59	<0.001
Outcome 3: Composite adverse outcome								
RNN (reference)	0.782 (0.761–0.802)	0.495 (0.452–0.538)	0.712	0.735	0.485	0.879	0.577	
GRU	0.835 (0.816–0.853)	0.582 (0.540–0.625)	0.775	0.784	0.552	0.91	0.645	<0.001
LSTM	0.868 (0.850–0.885)	0.648 (0.605–0.690)	0.815	0.806	0.596	0.928	0.689	<0.001

For diaphragmatic dysfunction (Outcome 1), LSTM demonstrated the highest discriminative ability (AUC: 0.845, 95% CI: 0.826–0.863; AUPRC: 0.594), indicating that longitudinal physiologic and ventilator trajectories contain useful early signals related to respiratory muscle dysfunction (Figure 4A). For composite adverse outcomes (Outcome 3), LSTM again performed robustly (AUC: 0.868; sensitivity: 81.5%), suggesting sensitivity to early deterioration patterns reflected in bedside monitoring data (Figure 4C). For high cognitive stress/delirium (Outcome 2), LSTM outperformed the baseline RNN (AUC: 0.792 vs. 0.715), although overall performance was lower than for the outcomes more directly tied to physiologic and ventilatory dynamics (Figure 4B). This pattern suggests that time-series monitoring signals alone may not fully represent evolving neurocognitive stress, motivating multimodal fusion with complementary inputs such as diaphragm ultrasound imaging.

### 3.5 Multimodal *versus* unimodal models for diaphragmatic dysfunction

**FIGURE 5:**
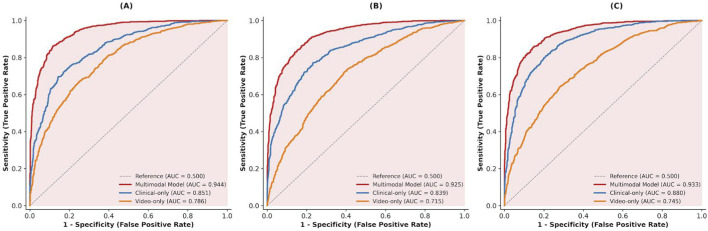
ROC curves of multimodal and ablation models for predicting diaphragmatic dysfunction. **(A)** Prediction of Diaphragmatic Dysfunction. **(B)** Predicion of High Cognitive Stress. **(C)** Prediction of Composite Outcome.

**FIGURE 6:**
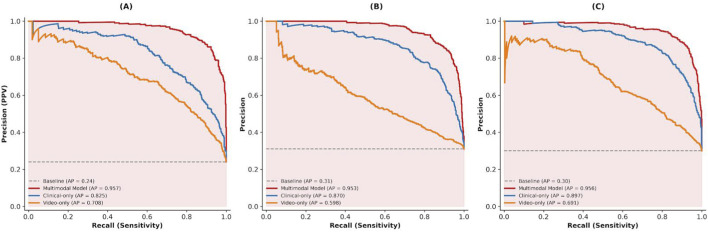
Precision–recall curves of multimodal and ablation models for predicting diaphragmatic dysfunction. **(A)** Diaphragmatic Dycfunction **(B)** High Cognitive Stress **(C)** Composite Adverse Outcome.

To quantify the incremental value of multimodal fusion, we compared the proposed multimodal model with two unimodal baselines: a clinical-only model using EHR-derived clinical time-series inputs and a video-only model using diaphragm ultrasound videos alone. ROC analyses (Figure 5) showed that the multimodal model achieved the best discrimination for diaphragmatic dysfunction (AUC = 0.902), significantly outperforming both the clinical-only model (AUC = 0.811; P < 0.001) and the video-only model (AUC = 0.749; P < 0.001). Precision–recall analyses (Figure 6) further indicated improved performance under class imbalance, with higher average precision and fewer false positives at comparable sensitivity.

**FIGURE 7:**
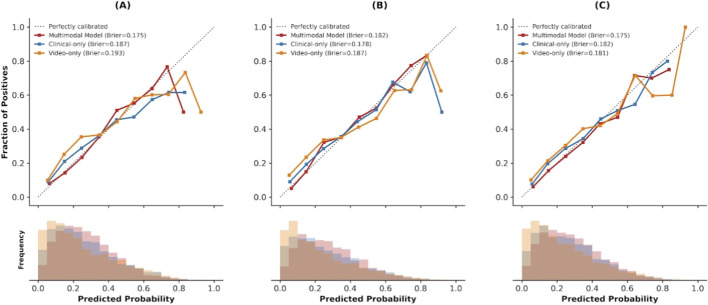
Calibration curves of multimodal and ablation models for predicting diaphragmatic dysfunction. **(A)** Diaphragmatic Dysfunction **(B)** High Cognitive Stress **(C)** Composite Adverse Outcome.

Calibration performance is shown in Figure 7. The multimodal model demonstrated close agreement between predicted probabilities and observed event rates (Brier score = 0.12), whereas the unimodal baselines showed systematic bias in high-risk ranges, with underestimation by the video-only model and overestimation by the clinical-only model.

**FIGURE 8:**
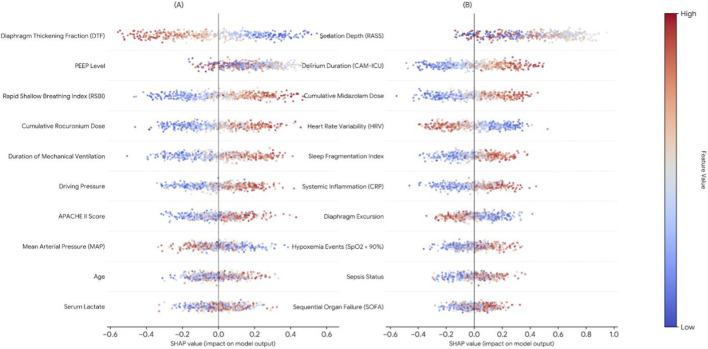
SHAP summary plot of the multimodal model highlighting key contributors to diaphragmatic dysfunction and cognitive stress prediction. **(A)** Predictors of Diaphragmatic dysfunction. **(B)** Predictors of high cognitive stress.

To examine which inputs contributed most to multimodal gains, we performed post-hoc SHAP analyses (Figure 8). The results suggested complementary contributions across modalities: ultrasound-derived DTF was among the most influential predictors, while cumulative neuromuscular blockade exposure and RSBI from clinical time-series data contributed additional discriminatory information.

### 3.6 Feature importance for diaphragmatic dysfunction

**FIGURE 9:**
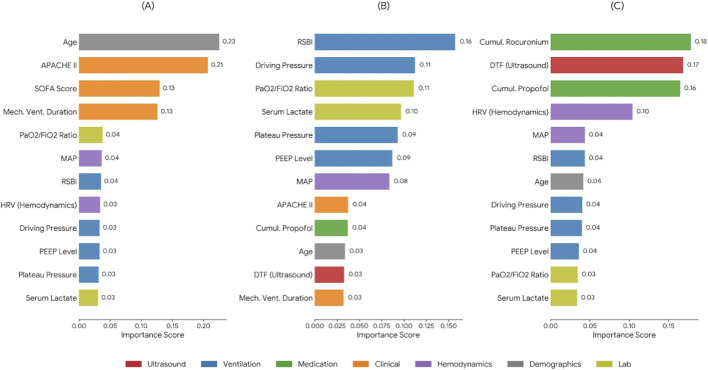
Variable importance generated by diaphragmatic dysfunction prediction models. **(A)** RNN importance. **(B)** GRU importance. **(C)** LSTM importance.

Global feature importance estimated by SHAP is shown in Figure 9. Ultrasound-derived DTF exhibited the highest relative importance (0.28), ranking first among all predictors, indicating that imaging-based diaphragm functional features provide strong predictive information for diaphragmatic dysfunction. Among clinical variables, RSBI (0.18) and cumulative rocuronium exposure (0.15) ranked as the second and third most influential predictors, respectively, exceeding the contribution of static severity scores such as APACHE II in this analysis.

Together, these rankings support a multifactorial risk profile for ventilator-associated diaphragmatic dysfunction, in which (i) imaging-derived diaphragm contractility (DTF), (ii) respiratory mechanics burden (RSBI), and (iii) iatrogenic neuromuscular suppression (neuromuscular blockade exposure) jointly contribute to elevated risk. The multimodal model appears to leverage nonlinear interactions across these domains to improve risk stratification.

### 3.7 Feature importance for cognitive stress

**FIGURE 10:**
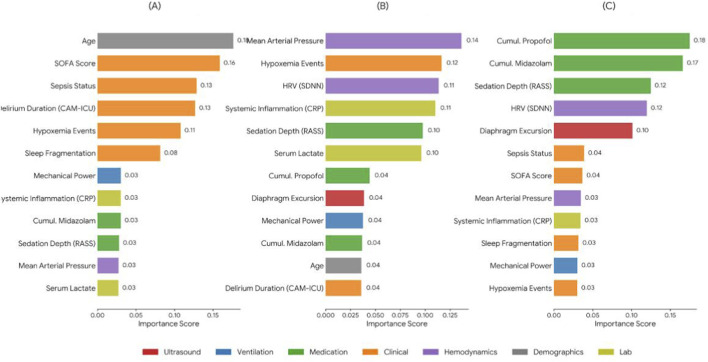
Variable importance generated by high cognitive stress prediction models. **(A)** RNN importance. **(B)** GRU importance. **(C)** LSTM importance.

To characterize drivers of high cognitive stress/delirium, we performed post-hoc SHAP analyses on the final multimodal model (Figure 10). The most influential predictors were sedation-management–related variables, with RASS and cumulative midazolam exposure ranking among the top contributors. This pattern is consistent with the clinically recognized role of prolonged and deep sedation in delirium risk and suggests that dynamic optimization of sedation depth and drug exposure may be a potentially modifiable leverage point.

Beyond sedation variables, the model highlighted two physiologically meaningful predictors with notable contributions: heart rate variability (HRV) and diaphragm excursion. Higher importance attributed to HRV suggests that autonomic dysregulation may precede or accompany neurocognitive stress trajectories in mechanically ventilated patients. Diaphragm excursion, derived from ultrasound imaging, also contributed substantially to cognitive stress prediction, indicating that respiratory mechanics and neurocognitive stress signals may co-evolve in a manner that benefits from multimodal integration. Collectively, these findings provide data-driven support for the hypothesized lung–brain crosstalk in ventilated ICU populations and help explain why time-aware multimodal models can outperform static or single-domain risk scoring approaches for neurocognitive outcomes.

## 4 Discussion

This multicenter study developed and validated an interpretable multimodal deep learning framework to jointly predict diaphragmatic dysfunction and high cognitive stress/delirium in mechanically ventilated ICU patients. By integrating diaphragm ultrasound video representations with longitudinal bedside physiologic trajectories and medication exposure patterns, the proposed approach achieved strong discrimination for diaphragmatic dysfunction and demonstrated clinically acceptable calibration. Importantly, multimodal fusion improved performance beyond clinical-only and video-only baselines, and SHapley Additive exPlanations (SHAP) provided transparent feature attributions to support clinician-facing interpretation.

Diaphragmatic dysfunction and neurocognitive stress are both common and clinically consequential complications in ventilated ICU patients, and their potential interrelationship has been increasingly discussed (Fang et al., 2025). Prolonged mechanical ventilation can promote diaphragm unloading and disuse atrophy and may co-occur with ICU-acquired weakness, undermining liberation success and prolonging ICU stay (Erbay Dalli and Kelebek Girgin, 2025). In parallel, high cognitive stress/delirium is associated with worse short-term outcomes and persistent cognitive sequelae after discharge (Vyveganathan et al., 2019). From a clinical workflow perspective, these risks are often assessed separately—diaphragm function through ultrasound and weaning indices, and neurocognitive status through screening tools—despite the fact that both trajectories are shaped by overlapping exposures such as ventilatory burden, sedation depth, and systemic illness severity (Abdelbaky et al., 2024). Our results support the feasibility of risk stratifying both functional endpoints within a unified framework, which better reflects how physiologic stressors accumulate and interact over time during mechanical ventilation.

A central finding is that multimodal fusion improved prediction of diaphragmatic dysfunction beyond unimodal baselines, with the multimodal model achieving an AUC of 0.902 in the independent test set. This gain is clinically plausible: diaphragm ultrasound videos encode spatiotemporal patterns of contractility and motion that extend beyond scalar summary parameters, while longitudinal clinical time-series and medication trajectories capture evolving ventilator settings, physiologic compensation, and cumulative iatrogenic exposures that ultrasound alone cannot contextualize (Song et al., 2025). In ICU environments where decisions depend on trends and accumulated burden rather than single-time-point observations, combining these complementary streams provides a more complete representation of patient state and helps reduce the brittleness of single-modality inference.

The explainability analyses offer additional clinical insight. For diaphragmatic dysfunction, SHAP highlighted ultrasound-derived measures of diaphragm function (e.g., DTF and diaphragm excursion), alongside variables reflecting respiratory mechanics and neuromuscular blockade exposure, as dominant contributors. For high cognitive stress/delirium, sedation depth and cumulative sedative exposure emerged as leading predictors, with physiologic features such as HRV and diaphragm excursion also contributing meaningfully. These patterns align with the clinical expectation that delirium risk is closely linked to sedation strategy and physiologic stress, while also suggesting that respiratory mechanics and autonomic dysregulation may co-evolve in a manner consistent with proposed lung–brain crosstalk. However, these signals should be interpreted as predictive associations rather than causal mechanisms: the model identifies combinations of features that discriminate risk trajectories, but it does not prove that modifying any single attributed factor will necessarily alter outcomes (Sato et al., 2025; Cavallazzi et al., 2012; Patel et al., 2023; Song et al., 2025). Nonetheless, the convergence of shared predictors across tasks provides a clinically grounded rationale for why integrated monitoring of respiratory mechanics and sedation-related physiology may support earlier recognition of high-risk trajectories, thereby enabling more proactive and individualized care.

This work extends prior ICU outcome prediction studies in three concrete ways. First, it addresses two clinically meaningful functional outcomes—diaphragmatic dysfunction and high cognitive stress/delirium—within a unified multimodal framework rather than treating them as independent single-outcome problems. Second, it leverages diaphragm ultrasound videos rather than relying solely on handcrafted ultrasound indices, enabling extraction of richer spatiotemporal representations that can complement structured bedside data. Third, by incorporating post-hoc interpretability through SHAP, the framework provides both global and patient-level feature attributions, which can improve transparency, support clinician trust, and facilitate hypothesis generation about potentially modifiable risk factors (Elfeky et al., 2025; Zhou L. et al., 2025; Fenske et al., 2025).

Several limitations warrant consideration. First, the retrospective design remains vulnerable to selection bias, site-specific practice patterns, and unmeasured confounding, even with multicenter data. Prospective evaluation will be needed to establish real-time performance and clinical utility under operational conditions. Second, outcome definitions in ICU settings are inherently challenging. Delirium assessments depend on documentation frequency and sedation depth, and diaphragm ultrasound measurements are influenced by acquisition quality and operator technique. Although this study used standardized protocols and excluded unassessable deep-sedation windows, misclassification and inter-site variation may persist. Third, SHAP provides additive attributions that improve interpretability, but post-hoc explanations do not guarantee mechanistic validity; attribution stability can also vary across correlated features and modeling choices, reinforcing the need for careful clinical interpretation before translating attributed predictors into intervention targets. Finally, standardizing diaphragm ultrasound acquisition and preprocessing across devices and centers remains a practical barrier to broad deployment, and future work on protocol harmonization and domain adaptation may be necessary to strengthen cross-site robustness (Ahmed et al., 2025).

Future studies should prospectively deploy this framework within ICU monitoring workflows to evaluate real-time alerting behavior, clinician usability, and downstream impact on ventilator management and neuroprotective strategies. Extension to additional ICU subpopulations and incorporation of other modalities—when available—may further improve robustness. Integration into clinical decision support systems (CDSS) should emphasize safety, interpretability, and clinician-centered workflow design, ensuring that model outputs augment rather than replace bedside judgment.

## Data Availability

The datasets used in this study are subject to institutional and ethical restrictions. All clinical time-series data and diaphragm ultrasound videos were obtained from real-world ICU patients and are protected under the approval of the Ethics Committee of the First Affiliated Hospital of Zhengzhou University (Approval No.: ZHZ-25W82). These data cannot be publicly released because they contain sensitive medical information and are prohibited from external sharing under hospital data-governance policies. Only de-identified data may be provided upon reasonable request to the corresponding author and contingent upon additional institutional approvals. Requests to access these datasets should be directed to JG: jinge2025123@outlook.com.
